# The 2015 Ming K Jeang Award for Excellence in *Cell & Bioscience*

**DOI:** 10.1186/s13578-016-0093-7

**Published:** 2016-04-19

**Authors:** Yun-Bo Shi

**Affiliations:** The National Institutes of Health, Bethesda, MD USA

## Abstract

Two research groups led by Dr. Bin Gao of National Institute on Alcohol Abuse and Alcoholism, National Institutes of Health, MD, USA and Dr. Keji Zhao of National Heart, Lung, and Blood Institute, National Institutes of Health, MD, USA, respectively, won the 2015 Ming K Jeang Award for excellence in *Cell & Bioscience.*

## Editorial

We are very pleased to announce that two research groups, who each published an outstanding research article in *Cell & Bioscience* in 2015, have been selected to receive the Ming K Jeang Award for excellence in *Cell & Bioscience*. The Ming K Jeang Award for excellence in *Cell & Bioscience* was established in 2011 with a generous donation from the Ming K. Jeang Foundation to honor outstanding research articles published in *Cell & Bioscience*, the official journal of the Society of Chinese Bioscientists in America (SCBA; www.scbasociety.org). A committee of *Cell & Bioscience* editors, chaired by Dr. Dong-Yan Jin, considered all research articles published in the journal in 2015 to select the following two articles to receive the award [1, 2]:

- *Biologically active, high levels of interleukin*-*22 inhibit hepatic gluconeogenesis but do not affect obesity and its metabolic consequences*
- Ogyi Park, Sung Hwan Ki, Mingjiang Xu, Hua Wang, Dechun Feng, Joseph Tam, Douglas Osei-Hyiaman, George Kunos and Bin Gao Cell & Bioscience 2015 5:25
- *Division of labor between IRF1 and IRF2 in regulating different stages of transcriptional activation in cellular antiviral activities*
- Gang Ren, Kairong Cui, Zhiying Zhang and Keji Zhao Cell & Bioscience 2015 5:17

Congratulations to these two groups of investigators for jobs well done!

We are looking forward to receiving contributions of outstanding research articles from the scientific community in 2016 and beyond.
